# Repurposing Clinical Drugs as AdoMetDC Inhibitors Using the SCAR Strategy

**DOI:** 10.3389/fphar.2020.00248

**Published:** 2020-03-11

**Authors:** Yan Zhang, Qiang Zheng, Yin Zhou, Sen Liu

**Affiliations:** ^1^National “111” Center for Cellular Regulation and Molecular Pharmaceutics, Key Laboratory of Industrial Fermentation (Ministry of Education), Hubei University of Technology, Wuhan, China; ^2^Institute of Biomedical and Pharmaceutical Sciences, Hubei Key Laboratory of Industrial Microbiology, Hubei University of Technology, Wuhan, China

**Keywords:** polyamines, drug repurpose, drug discovery, computer-aided drug design, AdoMet decarboxylase

## Abstract

With the escalating costs in drug development, discovering new uses of approved drugs, i.e., drug repurposing, has attracted increasing interest. Spermidine and spermine are important polyamines for most cells and their biosynthesis are strictly regulated by the polyamine metabolic network. In cancerous cells and tumor environments, the concentrations of polyamines are much higher than in normal cells. During the synthesis of spermidine and spermine, an amino-propyl group is provided by decarboxylated S-adenosylmethionine, and the latter is generated from S-adenosylmethionine by AdoMetDC (AdoMet decarboxylase). Therefore, as a rate-limiting enzyme in the biosynthesis of spermidine and spermine, AdoMetDC has been an attractive drug target in cancer studies. In the last decades, many AdoMetDC inhibitors have been discovered, and several AdoMetDC inhibitors are under clinical trials, but unfortunately, none of them have been approved yet. To overcome the high costs in time and money for discovering *de novo* inhibitors, we set out to repurpose clinic drugs as AdoMetDC inhibitors. We used steric-clashes alleviating receptors (SCAR), a computer-aided drug discovery strategy developed by us recently for *in silico* screening. By combining computational screening and experimental validation, we successfully identified two approved drugs that have inhibitory potency on AdoMetDC’s enzymatic activity. SCAR was previously shown to be suitable for the discovery of both covalent and non-covalent inhibitors, and this work further demonstrated the value of the SCAR strategy in drug repurposing.

## Introduction

Accompanying extended human lifespans and deteriorating environment, the world is facing increasing disease burdens from cancer, mental diseases, virus infections, etc. (Chang et al., 2019). Drugs have been proven to be indispensable for combating diseases and improving life quality, but the number of new drugs brought to the market has been declining (Scannell et al., 2012). One reason for this trend is that the costs of drug discovery have continued to increase. In the traditional drug development process, it costs over 15 years and one billion US dollars from target identification, target validation, hit discovery, and lead optimization to preclinical and clinical trials before a compound gets approved (Kaitin, 2010). Even more deadly to nascent pharmaceutical companies is that even if a compound enters phase II clinical trials, it has merely a 10% chance of getting approved due to unexpected human toxicity and lack of efficacy (Hodos et al., 2016). Therefore, saving costs in time, preclinical development, and even some or all clinical trials in drug development is extremely attractive, which is the main reason that drug repurposing (also known as drug repositioning, reprofiling, redirecting, or rediscovering) has attracted increasing attention in recent years (Baker et al., 2018). Drug repurposing identifies new uses for a drug beyond its original use, therefore, the data for human pharmacokinetics, safety, as well as the preclinical results, are readily available. This advantage makes it easier to get the new uses of the drug approved. Representative examples of drug repurposing include the sildenafil for erectile dysfunction and the anti-cancer use of thalidomide (Liu et al., 2013). Therefore, drug repurposing represents a most promising field in drug development (Cha et al., 2018).

Polyamines are small cationic compounds that exist in nearly all cells (Miller-Fleming et al., 2015). In mammalian cells, the most common polyamines are putrescine, spermidine, and spermine. The structures of these polyamines are rather simple, with linear aliphatic chains bridged or flanked by amino groups, however, they can affect the structures and functions of nucleic acids, proteins, and membranes (Miller-Fleming et al., 2015). Therefore, polyamines are indispensable players in regulating cellular signaling modules and cell fates. The cellular concentrations of these polyamines could be up to 20 mM (Li et al., 2019), which is strictly tuned by a complicated metabolic network including biosynthesis, interconversion, metabolism, and membrane transportation (Pegg, 2009). It has been well demonstrated that the proliferation of cancer cells requires higher cellular polyamine levels than normal cells, so the polyamine metabolic network in cancer cells is dysregulated to increasing the biosynthesis or membrane uptake of these polyamines (Casero, 2011). Consequently, targeting the polyamine metabolic network to inhibit the biosynthesis and/or membrane uptake of polyamines is a promising anti-cancer strategy (Casero, 2011).

**FIGURE 1:**
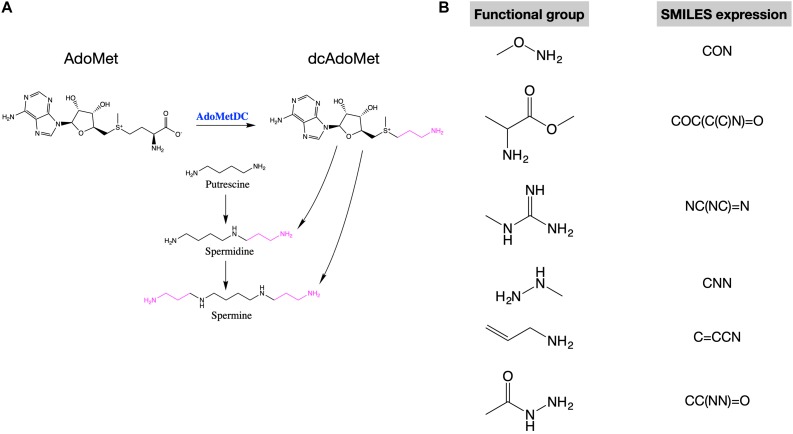
AdoMetDC is a rate-limiting enzyme for polyamine synthesis and several functional groups repeatedly occur in AdoMetDC inhibitors. **(A)** The reaction catalyzed by AdoMetDC is from AdoMet to decarboxylated AdoMet (dcAdoMet), which provides the amino-propyl group for the biosynthesis of spermidine and spermine. **(B)** The functional groups observed in previous AdoMetDC inhibitors were used for *in silico* screening in this study. The SMILES expressions used for library filtering are shown next to the structure.

In mammalian cells, putrescine is the precursor of spermidine and spermidine is the precursor of spermine. During these conversions, an amino-propyl group from decarboxylated S-adenosylmethionine (dcAdoMet) is transferred (Figure 1A). The production of dcAdoMet is generated from the decarboxylation of S-adenosylmethionine (AdoMet), which is catalyzed by AdoMetDC (AdoMet decarboxylase) (Figure 1A). Therefore, AdoMetDC is a rate-limiting enzyme in the biosynthesis of polyamines (Liao et al., 2015). To inhibit polyamine synthesis, many AdoMetDC inhibitors have been developed, including the first-generation inhibitor methylglyoxal bis (guanylhydrazone) (MGBG), the second-generation inhibitor SAM486A (Sardomozide, also known as CGP48664), and the third-generation inhibitor AbeAdo (5′-([(Z)-4-amino-2-butenyl]methylamino)-5′-deoxy- adenosine), etc. (Casero, 2011). Several AdoMetDC inhibitors have been tested in clinical trials treating cancers (Herr et al., 1986; Paridaens et al., 2000), but none of them has been finally approved for clinical use due to low efficiency or strong side-effects. Therefore, discovering new AdoMetDC inhibitors will be highly valuable.

Recently, we described the computer-aided discovery of both non-covalent and covalent AdoMetDC inhibitors based on the *in silico* protein design strategy (Liao et al., 2015; Ai et al., 2016). To assist the discovery of covalent AdoMetDC inhibitors, we presented a docking strategy named as steric-clashes alleviating receptors (SCAR) (Ai et al., 2016). Additionally, we demonstrated that this strategy also works good for evaluating non-covalent inhibitors (Ai et al., 2016). In this work, we set out to test the use of the SCAR method in repurposing the drugs in clinical trials as AdoMetDC inhibitors. We successfully identified two drugs worth further investigation. Our results showed that the SCAR method is an attracting strategy for both *de novo* drug discovery and drug repurposing.

## Materials and Methods

### Structure Preparation of the Small Molecules

The 3D structures of the small molecules were downloaded as mol2 files from ZINC15^1^ (Sterling and Irwin, 2015). In ZINC15, the 3D conformations were protonated at physiological pH and biologically relevant tautomers were generated for each molecule, which resulted in more than one conformation for many molecules. From these mol2 files, MGLTools (version 1.5.6) was used to generate the PDBQT files for docking.

### Structure Preparation of the Protein

The AdoMetDC structure (PDB ID: 3DZ5) was downloaded from PDB^2^. The Pyr68 residue of the alpha-chain of AdoMetDC was eliminated as previously mentioned (Ai et al., 2016) before the structure was energetically minimized using the relax protocol in Rosetta (Leaver-Fay et al., 2011). The options for minimization were: -relax:constrain_relax_to_start_coords -relax:coord_constrain_sidechains -relax:ramp_constraints false -s protein.pdb -ex1 -ex2 -use_input_sc -flip_HNQ -no_optH false. Finally, MGLTools (version 1.5.6) was used to generate the PDBQT file for docking.

### *In silico* Docking

The computational docking of the small molecules to AdoMetDC was similar with the process previously described (Ai et al., 2016). Briefly, the substrate binding pocket of AdoMetDC was used as the reference to define a grid box in MGLTools. Then, AutoDock Vina (version 1.1.2) was used to dock small molecules to the indicated grid box of AdoMetDC. The exhaustiveness parameter was set as 100 to extensively search possible docking conformations. For each ligand, up to 20 conformations were output. Finally, the docked conformations were then ranking by docking scores. After docking, we used the following rules to filter the docking results: (1) The RMSD cutoff was set as 3.0 compared to the first conformation, (2) the score cutoff was set as −8.0 (Ai et al., 2016), and (3) for each compound, 75% or more output conformations fulfilled these two cutoffs. Additionally, to minimize the numbers of the out-of-pocket atoms, an affinity density was defined as the average score of the non-hydrogen atoms in the compound and set as −0.28 (lower is better). After that, the docked conformations were manually checked to evaluate the structural similarity and the position of the functional groups.

### Compounds and Materials

The compounds used for experimental screening were purchased from the following commercial vendors: ZINC-000001530713 and ZINC-000006482036 from MedChemExpress^3^; ZINC-000043195697 from Selleck Chemicals^4^; ZINC-000043205655, ZINC-000100055899, and ZINC-000144542146 from Topscience^5^. The compounds were fully dissolved in DMSO to prepare 60 mM stock solutions. The carbon dioxide kit was purchased from BioSino Bio-Technology and Science Inc. (Beijing, China).

### Protein Expression and Purification

The expression and purification of human AdoMetDC were similar as mentioned before (Liao et al., 2015; Ai et al., 2016). Briefly, the coding sequence of AdoMetDC was inserted in pET15b, and the plasmid was transformed into the *E*scherichia *coli* strain BL21(DE3). The expression of AdoMetDC was induced by 0.5 mM of IPTG (isopropyl β-d-1-thiogalactopyranoside) at 15°C, 250 rpm for 12 h. The cells were collected, resuspended, and broken by high-pressure homogenizer in the cell lysis buffer (20 mM Na_2_HPO_4_, 500 mM NaCl, pH 7.0, 2.5 mM putrescine, 30 mM imidazole). The solution was centrifuged, and the supernatant was loaded in a Ni-NTA gravity column for the purification of His-tagged AdoMetDC. Finally, the protein was purified with a Sephacryl S-200 HR column (GE Healthcare) and eluted with the elution buffer (300 mM NaCl, 0.1 mM EDTA, 2.5 mM DTT, 1 mM HEPES, pH 7.0). The purified AdoMetDC was used for the activity analyses without removing the His tag.

### AdoMetDC-PEPC-MDH Assay

The AdoMetDC-PEPC-MDH assay was developed in our previous study (Liao et al., 2015) and used to evaluate the activity of AdoMetDC with the procedures similar as before (Liao et al., 2015; Ai et al., 2016). Briefly, AdoMetDC was mixed with DMSO, MGBG or compounds, respectively, in wells and incubated at 37°C for 30 min before R2 [400 unit/L phosphoenolpyruvate carboxylase (PEPC), 600 unit/L malate dehydrogenase (MDH), 0.45 mM NADH] was added to the wells in a 96-well plate. In another row, R1 [7.0 mM phosphoenolpyruvate (PEP), 8.0 mM MgCl_2_] and S-adenosylmethionine (AdoMet) were mixed. The plate was incubated in a 37°C incubator for 5 min, and the reaction was initiated by mixing the two solutions in these two wells. The absorbance data at 340 nm was recorded every 30 s at 37°C on a multifunctional microplate reader (BioTek Synergy H1) for 10 min. The final reaction mixture contained 120 μL R1, 40 μL R2, 1 mM AdoMet, 1 μM AdoMetDC, and 100 μM MGBG or compounds. For each compound, at least three independent experiments were performed.

### HPLC (High Performance Liquid Chromatography) Assay

The compounds were incubated with AdoMetDC in the reaction buffer (20 mM Na_2_HPO_4_, pH 7.0, 100 mM NaCl) at 37°C for 30 min. AdoMet was added to initiate the reaction. The final reaction volume was 200 μL, containing 100 μM inhibitors, 1 μM AdoMetDC, and 1 mM AdoMet. At the indicated time points, the reaction was stopped by 800 μL of methanol. The samples were centrifuged at 20,000 *g* for 5 min, and the supernatants were analyzed. The analysis was performed using a reverse-phase column (Waters C18 column, 5 μm, 4.6 × 250 mm) on a Dionex Ultimate 3000 system (Thermo Scientific) at 30°C, 254 nm. The mobile phase was 10 mM ammonium format (pH 3.5, adjusted with formic acid) and methanol (97:3, v/v), and the flow rate was 0.7 mL/min. The data were collected and the peak areas were integrated in the Chromeleon software. For each compound, at least three independent experiments were performed.

### Mass Spectrometry Analysis

The compounds were used for experimental screening. Samples were prepared and analyzed following the previous method (Ai et al., 2016). Briefly, the inhibitors were incubated with AdoMetDC in the reaction buffer (20 mM Na_2_HPO_4_, pH 7.0, 100 mM NaCl) at 37°C for 120 min. The final reaction volume was 200 μL, including 100 μM inhibitors and 10 μM AdoMetDC. The matrix-assisted laser desorption ionization time-of-flight mass spectrometry (MALDI-TOF-MS) analysis was performed on an Applied Biosystems 5800 (AB SCIEX, Concord, Canada) equipped with a 355 nm Nd:YAG laser source. SA matrix (sinapic acid) was prepared in 30% acetonitrile (ACN) aqueous solution. The acquired data were processed in AB Sciex Data Explorer v4.5 (AB SCIEX, Concord, Canada). For each compound, at least three independent experiments were performed.

## Results

### Database Preparation for *In silico* Docking

We were interested in investigating if any in-trial drugs are potential AdoMetDC inhibitors. Therefore, we obtained the in-trials compound library from the ZINC database^6^, which contains 5,811 compounds. Among these compounds, 3,447 are approved drugs by the Food and Drug Administration (FDA) of United States or other major juridications, and another 2,364 compounds are drugs in clinical trials (Sterling and Irwin, 2015). A subset docking library was prepared from this compound library by filtering the compounds with the functional groups of AdoMetDC inhibitors defined in our previous work (Ai et al., 2016). Both non-covalent and covalent functional groups were used in this step (Figure 1B), since we wanted to search for both covalent and non-covalent AdoMetDC inhibitors. The pre-defined functional groups were represented by their simplified molecular input line entry system (SMILES) expressions as below: CON, COC(C(C)N) = O, NC(NC) = N, CNN, C = CCN, CC(NN) = O. Finally, 613 compounds were included in the docking library. Furthermore, the different conformations of these compounds were downloaded from ZINC15 as is (Sterling and Irwin, 2015), resulting in a docking library containing 1,074 entities.

### SCAR Screening of Potential AdoMetDC Inhibitors

**FIGURE 2:**
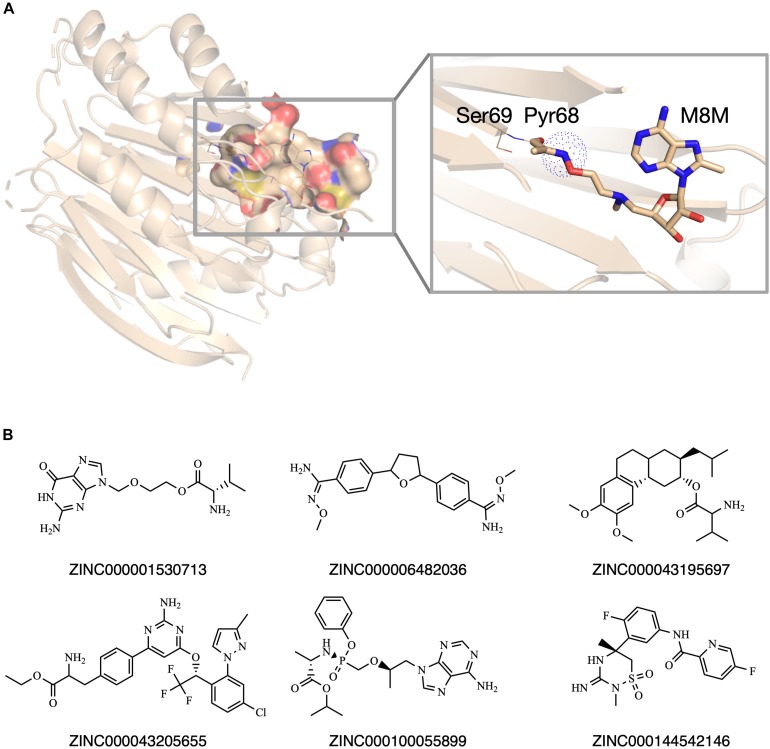
The *in silico* screening of potential AdoMetDC inhibitors. **(A)** The covalent inhibitor M8M is covalently attached with Pyr68 in AdoMetDC (PDB ID: 3DZ5). For SCAR docking, the Pyr68 of AdoMetDC was computationally removed, and the dotted sphere was defined as the covalent center to evaluate if a ligand is a potential covalent inhibitor as previously described (Ai et al., 2016). **(B)** Six compounds were chosen from the *in silico* screen for experimental validation. The ZINC IDs were shown below the corresponding structures. All structural figures in this paper were prepared in Pymol.

Previously, our group set up a SCAR strategy for screening covalent AdoMetDC inhibitors (Ai et al., 2016). As demonstrated in the previous work (Ai et al., 2016), this method was able to recapture the X-ray conformations of both covalent and non-covalent AdoMetDC inhibitors. Therefore, we adopted this method for *in silico* screening in this work. Similar as described in our previous work (Ai et al., 2016), a high-resolution AdoMetDC structure (PDB ID: 3DZ5) was used as the target. To perform SCAR docking, the Pyr68 residue of the alpha-chain of AdoMetDC was wholly eliminated (Figure 2A), and the structure was energetically minimized in Rosetta (Leaver-Fay et al., 2011). The substrate binding pocket in AdoMetDC was defined as the search region, and the compound entities in the prepared docking library were docked to this pocket one by one. Following the docking process, the candidate ligands were evaluated by the rules as described in Materials and Methods. At last, six compounds were chosen and available for purchase to do experimental validation (Figure 2B). Meanwhile, SAM486A was also identified as a candidate during this computational process, but it was not included in further experimental tests since it is a known AdoMetDC inhibitor.

### Experimental Validation of Potential AdoMetDC Inhibitors

**FIGURE 3:**
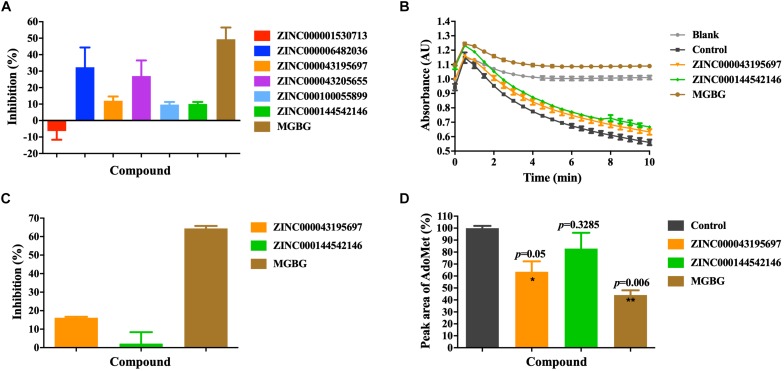
The experimental validation of the inhibitory efficacy of the purchased compounds. **(A)** The inhibitors were evaluated by the AdoMetDC-PEPC-MDH assay (Liao et al., 2015) quantifying the released CO_2_ from decarboxylation. The absorbance changes (ΔAU) in the first 5 min were used to calculate the inhibition percentages compared to the control without inhibitors. A note is although ZINC6482036 and ZINC43205655 showed high inhibition percentages in this assay, they were found to be false positives in the HPLC assay. **(B)** ZINC000043195697 and ZINC000144542146 were tested in the second-phase evaluation with the AdoMetDC-PEPC-MDH assay. **(C)** The calculated inhibition rates from the data in **(B)**. **(D)** The inhibitors were evaluated by the HPLC assay quantifying the consumption of AdoMet during the reaction. The amounts of AdoMet were quantified by calculating the peak areas and normalized against the control. The control was the sample without AdoMetDC added. All compounds were tested at 100 μM. The data are shown as mean ± SEM (*n* = 3), and the Student’s *t*-test was performed for statistical analysis. *p ≤ 0.05; **p ≤ 0.01.

We used the AdoMetDC-PEPC-MDH assay developed in our previous study (Liao et al., 2015) to experimentally validate the inhibitory potency of the purchased compounds on AdoMetDC’s enzymatic activity. This assay quantifies the generation of CO_2_ from the decarboxylation reaction and is suitable for the initial semi-quantitative screening. As shown in Figure 3A, among the six compounds, ZINC000043195697 and ZINC000144542146 slightly inhibited AdoMetDC’s activity in the first-round fast screening. As of ZINC6482036 and ZINC43205655, we noticed that their light absorbance values were distorted by their low solubility and their inhibitory effects were not reliable (data not shown). This was not unexpected since the AdoMetDC-PEPC-MDH assay is fast but prone to interferences. Next, a more careful evaluation was repeated to confirm the inhibitory potency of ZINC000043195697 and ZINC000144542146. The data (Figures 3B,C) showed that ZINC000043195697 was better than ZINC000144542146, although they were both less potent than MGBG.

Following the quick screening step using the AdoMetDC-PEPC-MDH assay, we continued to verify the inhibitory potency of ZINC000043195697 and ZINC000144542146 more precisely. To this end, we performed an HPLC assay, which quantifies the consumption of the substrate AdoMet during the reaction. The HPLC result (Figure 3C) confirmed that ZINC000043195697 obviously inhibited AdoMetDC’s activity and ZINC000144542146 could not significantly inhibit AdoMetDC.

### Analysis of the Binding Structure and Mechanism

**FIGURE 4:**
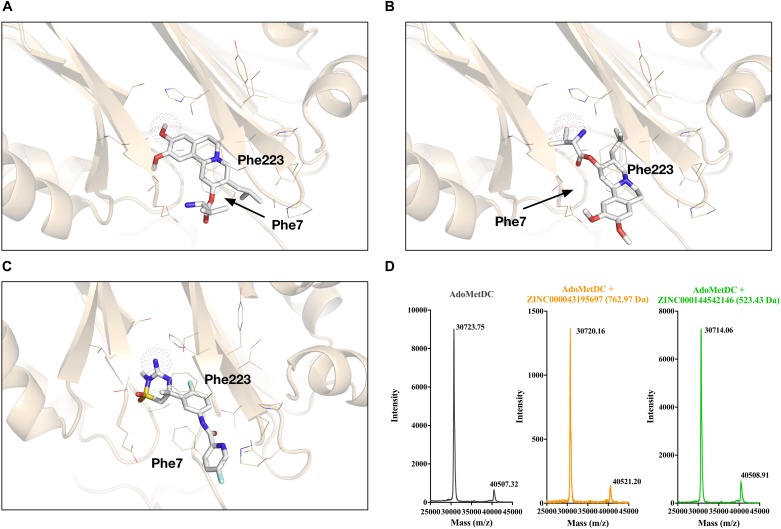
Analyses of the binding conformations and the binding mechanisms of the identified AdoMetDC inhibitors. **(A)** The overall best (1st) docking conformation of ZINC000043195697 with AdoMetDC. **(B)** The best (4th) docking conformation of ZINC000043195697 with an amino group close to the covalent center (shown in dotted sphere). **(C)** The overall best (1st) docking conformation of ZINC000144542146 with AdoMetDC. This is also the best docking conformation with an amino group close to the covalent center. **(D)** The mass spectrometry (MS) data used to evaluate the covalent binding of the inhibitor and AdoMetDC. The molecular weights of the compounds are shown in parentheses.

To analyze the binding mechanism of the identified inhibitors with AdoMetDC, we looked into the docked conformations. For ZINC000043195697, the first docking conformation is shown in Figure 4A and the best docking conformation with a possible functional amino group close to the covalent center is shown in Figure 4B. Both poses form preferred π–π and/or cation–π interactions with Phe7 and Phe223 of AdoMetDC (Ai et al., 2016). However, the first pose (Figure 4A) has a hydroxyl group that will conflict with Pyr68 (not shown) since this group cannot form covalent bonds with Pyr68. For the second pose (Figure 4B), the amino group is not placed very well in the covalent sphere, so this might affect the formation of covalent bonds and the binding affinity. For ZINC000144542146, the first docking conformation is shown in Figure 4C, which was also a conformation with a potential functional amino group in the covalent sphere. The backbone phenyl ring also forms π–π interactions with Phe7 and Phe223 of AdoMetDC. Therefore, we continued to test if these two compounds could covalently bind AdoMetDC. As previously described (Ai et al., 2016), a MALDI-TOF-MS analysis was performed. However, the data (Figure 4D) showed that no ligands were attached to the protein and caused significant increases in the mass of either the alpha chain (theoretical Mw: 30655 Da) or the whole protein (theoretical Mw: 40635 Da), indicating that these compounds should not be AdoMetDC’s covalent inhibitors.

## Discussion

Drug repurposing holds high promise in lowering the economic burden and increasing the success rate in drug development. Both experimental screening approaches and *in silico* screening approaches are useful in drug repurposing (Cha et al., 2018), but *in silico* approaches are more economic and could be highly efficient in enriching candidate drugs for experimental validation (Battah et al., 2019). In this work, we extended our SCAR method to the repurposing of clinical drugs as AdoMetDC inhibitors. As demonstrated above, we were able to identify two drugs that can inhibit AdoMetDC’s enzymatic activity, which indicates that our screening protocol was successful. However, the false positive signals of ZINC6482036 and ZINC43205655 suggested that the AdoMetDC-PEPC-MDH assay should be performed carefully and coupled with more precise experimental assays such as the HPLC assay.

Two aspects of our *in silico* screening process proved the high potential of the SCAR strategy in drug repurposing. Firstly, we were able to successfully screen out the known AdoMetDC inhibitor, SAM486A (also known as ZINC000100023874, Sardomozide, or CGP48664) (Pless et al., 2004), from the database. In fact, we used a blind test during the screening process: both the researchers performing the docking process and the researchers manually shortlisting the candidate drugs were not aware of the existence of SAM486A in the database. However, following the rules in this work, this compound was robustly identified by different researchers. Secondly, we successfully validated the inhibitory effects of two compounds in inhibiting AdoMetDC’s activity from six compounds. This successful ratio is quite encouraging for the future application of the SCAR strategy on other targets.

As frequently noticed in previous studies, one of the main drawbacks of drug repurposing is that the drug usually has only moderate or very low activity against the new targets (Zheng et al., 2018). This is also true in this work, since the identified compounds only showed moderate to low activities in inhibiting AdoMetDC’s activity. One possible reason is the functional groups in ZINC000043195697 and ZINC000144542146 are not active enough to form covalent bonds with Pyr68 of AdoMetDC, so the steric clashes decrease their binding affinities. Nonetheless, there are two possible solutions for this issue. One solution is to use drug combination to increase the potency of this drug (Zheng et al., 2018). For example, combining the identified inhibitors with the inhibitors of polyamine transportation (such as AMXT-1501) to lower the effective concentration of the AdoMetDC inhibitors (Gamble et al., 2019). Another solution is to optimize the structure of the identified compounds to improve their activity. However, this solution would indicate more intensive investigations.

Among the two identified AdoMetDC inhibitors, ZINC000043195697 is valbenazine, which is a vesicular monoamine transporter 2 (VMAT2) inhibitor. Valbenazine is the first FDA approved drug for adults with tardive dyskinesia and sold under the trade name Ingrezza. The other one is verubecestat (ZINC000144542146), an inhibitor of beta-secretase 1 treating Alzheimer’s disease. Considering that polyamines play important roles in neurodegeneration diseases (Cervelli et al., 2014), their potential role in inhibiting AdoMetDC might be worth further investigations to understand their physiological roles as well as side-effects. For instance, both valbenazine (Thai-Cuarto et al., 2018) and verubecestat (Egan et al., 2018) were reported to cause imbalance and falls in the patients, so if this symptom has any relationship with the change of polyamine levels is an interesting question, considering that polyamine levels are decreasing with aging (Minois et al., 2011) but imbalance is usually increasing with aging.

## Conclusion

In conclusion, our work demonstrated that, in addition to being useful in *de novo* drug screening (Ai et al., 2016), the SCAR strategy is also applicable in drug repurposing. Although this work did not find covalent candidates due to the limitations of the database, we hope the SCAR method will find more applications in repurposing both non-covalent and covalent drugs in the future.

## Data Availability Statement

The datasets generated for this study are available on request to the corresponding author.

## Author Contributions

SL conceived the idea. SL and QZ did the computational work. SL and YaZ designed the experiments. YaZ did the experimental work. SL, QZ, and YaZ analyzed and interpreted the data. SL, YaZ, and YiZ wrote the manuscript. All authors reviewed and approved the submitted manuscript.

## Conflict of Interest

The authors declare that the research was conducted in the absence of any commercial or financial relationships that could be construed as a potential conflict of interest.

## Footnotes

1 http://zinc15.docking.org

2 http://www.rcsb.org

3 https://www.medchemexpress.com

4 http://www.selleckchem.com

5 http://www.topscience.cn

6 http://zinc15.docking.org
